# Reference Intervals of Selected Serum and Plasma Biochemical Analytes in Clinically Healthy Multiparous Holstein Cows During the Periparturient Period

**DOI:** 10.1111/vcp.70044

**Published:** 2025-09-29

**Authors:** Nektarios Siachos, Georgios Oikonomou, Nikolaos Panousis, Ioannis Sampsonidis, Stavros Kalogiannis, Georgios Arsenos, Georgios E. Valergakis

**Affiliations:** ^1^ Laboratory of Animal Husbandry, Faculty of Veterinary Medicine, School of Health Sciences Aristotle University of Thessaloniki Thessaloniki Greece; ^2^ Department of Livestock and one Health, Institute of Infection, Veterinary & Ecological Sciences, Faculty of Health and Life Sciences University of Liverpool Neston UK; ^3^ Clinic of Farm Animals, Faculty of Veterinary Medicine, School of Health Sciences Aristotle University of Thessaloniki Thessaloniki Greece; ^4^ Department of Nutritional Sciences and Dietetics International Hellenic University Thessaloniki Greece

**Keywords:** dairy cattle, energy balance, hepatic function, protein metabolism, renal function, transition period

## Abstract

**Background:**

Monitoring the metabolic changes during the periparturient period is crucial.

**Objective:**

We aimed to establish reference intervals (RIs) of several serum and plasma biochemical variables in multiparous periparturient Holstein dairy cows.

**Methods:**

A total of 238 multiparous dry cows from six commercial dairy farms were enrolled. Blood samples were collected at predetermined time points (21 and 7 days before the expected calving date, 7, 21, and 28 days after calving, ±2 days on all occasions). Samples from 113 cows that met the inclusion criteria were analyzed for plasma and serum biochemical analytes.

**Results:**

The lower and upper reference limits were 40.9–49.3 and 102.8–128.5 U/L, respectively, for aspartate aminotransferase; 9.1–14.0 and 33.8–47.8 U/L for γ‐glutamyl transferase; 0.07–0.13 and 0.20–0.43 mmol/L for triglycerides; 1.11–1.25 and 3.04–3.52 mmol/L for cholesterol; 0.0 and 3.42–7.78 μmol/L for total bilirubin; 141.0–285.0 and 778.6–1279.3 μmol/L for β‐hydroxybutyrate, 0.07–0.22 and 0.42–1.24 mmol/L for non‐esterified fatty acids; 50.8–59.7 and 77.4–103.5 g/L for total protein; 23.6–29.2 and 39.4–48.5 g/L for albumin; 19.4–28.3 and 42.0–58.3 g/L for globulin; 0.59–0.70 and 1.30–1.61 for albumin/globulin ratio; 1.3–3.2 and 7.6–9.7 mmol/L for blood urea nitrogen; 42.4–62.8 and 83.1–124.7 μmol/L for creatinine; 1.8–3.2 and 9.8–25.1 μmol/L for 3‐methylhistidine; and 1.9–2.7 and 10.5–14.8 μmol/L for 1‐methylhistidine.

**Conclusions:**

The established RIs provide valuable benchmarks with important clinical and research applications in dairy cattle medicine.

## 1 Introduction

The transition period in dairy cows, usually spanning 3 weeks before and three or 4 weeks after calving, involves significant physiologic changes that influence overall health and productivity [1]. To support lactation, transition cows undergo metabolically intense homeorhetic adjustments, which, when exacerbated, can lead to health issues [1, 2]. Monitoring blood biochemical parameters during this period can offer useful insights into metabolic status, facilitating prevention, early detection, and management of health and reproductive issues [3, 4].

Reference intervals (RIs) are essential for interpreting laboratory results and guiding clinical decisions [5, 6]. Quiroz‐Rocha et al. [7] established RIs for several serum parameters in Canadian dairy cows within 1 week before and after calving. Brscic et al. [8] established RIs for blood parameters in late‐pregnant heifers and dry cows, considering the effects of parity, days relative to calving, and season. Moretti et al. [9] provided RIs for hematological and biochemical parameters in Holstein cows around 3 and 30 days after calving, highlighting the changes in acute phase proteins and markers of oxidation. Cozzi et al. [10] explored reference values, reported as mean values and confidence intervals, for several biochemical parameters in lactating dairy cows, accounting for the effect of parity, lactation stage, and season. The biochemical analytes comprising the metabolic profile in dairy cows have been described and categorized based on their relevance to energy metabolism, protein balance status, liver and kidney function, and overall metabolic health, while some overlap between categories exists [3, 4].

Despite the importance of metabolic profiling during the transition period [4], previous studies are rather inconclusive considering the established RIs and the fact that health monitoring of cows occurred only for parts of this period. Moreover, there is a lack of established RIs for 3‐methylhistidine (3‐MH) and 1‐methylhistidine (1‐MH) that are recognized by recent research as key biochemical parameters [4, 11]. In dairy cattle, 3‐MH, also known as τ‐methylhistidine, results from the breakdown of myosin and actin and is regarded as a reliable indicator of skeletal muscle protein turnover [4, 11]. 1‐MH, also known as π‐methylhistidine, is another methylated derivative of histidine produced from the breakdown of anserine, a dipeptide found in muscle tissue [11, 12]. Both isomers are not recycled in the body and are recovered in comparable concentrations [12].

Hence, the objective here was to establish RIs for several serum and plasma biochemical analytes indicative of energy balance, liver health, protein metabolism, and renal function in clinically healthy multiparous Holstein dairy cows during the whole periparturient period.

## 2 Materials and Methods

The study was conducted in compliance with institutional and ethical guidelines set by the Research Committee of the Aristotle University of Thessaloniki, Greece, and was approved by the Review Board (Approval Protocol Number: 62/15‐12‐2015). Farmers gave informed consent for their animals to be included in the study.

Data were collected from September 2016 to October 2019 during a prospective cohort study investigating the mobilization of skeletal muscle and subcutaneous fat reserves as well as the evolution of protein metabolism in periparturient Holstein cows. In total, 238 multiparous cows from six commercial dairy farms were selected at Day 21 prior to the expected calving day and were continuously monitored until 28 days after calving. Cows in all participating farms were housed indoors, following a year‐round calving pattern, and were fed total mixed rations formulated to meet or exceed nutritional requirements. Farms and cows, respectively, were enrolled consecutively in the study. The first author (qualified veterinarian) visited the farms three times per week for animal measurements and data collection to ensure close monitoring. Cows were examined at predetermined time points covering the whole transition period as follows: −21, −7, 0, 7, 21, and 28 days (0d defined as the day of calving; ±2 days in all cases). Cows were first assessed for their body condition score (BCS) using the 1–5‐point scale with 0.25‐unit increments [13]. Thereafter, a clinical examination was performed as follows: (a) recording of rectal temperature with a digital thermometer; (b) estimation of rumen fill score [14]; (c) auscultation of the rumen for 2 min at the left paralumbar fossa [15] and recording the number of rumen contractions; (d) lung auscultation and recording of respiratory rate; (e) heart auscultation and recording of heart rate, and (f) assessment of hydration status. At 0, 7, 21, and 28 days, all cows were additionally examined for specific clinical signs of retained fetal membranes, milk fever, metritis, mastitis, ketosis, right or left displacement of the abomasum, and pneumonia. Any clinical cases occurring between the study days were diagnosed by the farm veterinarians or farm staff using predefined protocols and were then confirmed by the first author. Following the clinical examination and after being released from the headlocks, locomotion of the cows was assessed using the 1–5 grade scoring method [16].

The inclusion and exclusion criteria for the computation of the RIs were:

- Cow presence at the farm until the end of the study (no death or culling).
- Normal rectal temperature at any time‐point [cows with fever (> 39.1°C) or hypothermia (< 38.0°C) were excluded].
- Normal rumen function (cows with a rumen fill score < 2 and/or with < 1 rumen contractions/2 min were excluded), at any time point except at 0 day.
- Exclusion of any cow that required human assistance or intervention during calving.
- Exclusion of any cow with clinical signs of any of the aforementioned clinical diseases at any time point.
- Exclusion of any cow with a locomotion score < 3, at any time point.

Two blood samples were collected by coccygeal venipuncture using 18 G × 25 mm needles from each cow at −21, −7, 7, 21, and 28 days. The first blood sample was collected into a 6 mL sterile glass vacuum tube with Lithium‐heparin as anticoagulant (BD Vacutainer, Plymouth, UK), and the second into a 10 mL sterile glass vacuum tube without anticoagulant (BD Vacutainer, Plymouth, UK). Blood sampling was consistently conducted after the morning milking, between 08:00 and 10:00 across all farms, to ensure that measurements are not influenced by diurnal variation. Blood samples were placed in a rack inside a portable cooler with ice immediately after collection and transported to the lab within 2 h. Plasma and serum were harvested following centrifugation (3,000 *g* × 15 min, at room temperature) and stored in 1.5 mL polyethylene tubes at −20°C pending analysis. A total of six analytical runs were performed, with samples stored at −20°C for 1–4 months before analysis, following the completion of sampling on each farm.

Serum samples were analyzed by photometry using the automated Siemens ADVIA 1800 chemistry system to determine the activity of aspartate aminotransferase (AST) and γ‐glutamyl transferase (GGT), and the concentration of triglycerides (TRIG), cholesterol (CHOL), total bilirubin (tBIL), β‐hydroxybutyrate (BHB), non‐esterified fatty acids (NEFA), total protein (TP), albumin (ALB), blood urea nitrogen (BUN), and creatinine (CRE). The method for each assay, along with the intra‐ and inter‐assay coefficient of variation and the corresponding mean concentrations for each analyte, is presented in Table 1. Intra‐assay repeatability was determined by measuring the biochemical analytes in the same Quality Control Material (QCM) sample 10 times sequentially within a single assay run. Inter‐assay repeatability was determined by analyzing the same QCM sample once daily over 10 separate days. Globulin serum concentration was calculated by subtracting the ALB fraction from TP. The ADVIA 1800 analyzer automatically evaluates haemolysis, icterus, and lipemia for each sample during preanalytical quality control, and samples indicating any of these conditions were excluded from the analysis.

**TABLE 1:** Method, intra‐ and inter‐assay coefficients of variation (CV) and corresponding mean concentrations for determining the concentration of serum biochemical analytes, by photometry using the Siemens ADVIA 1800 chemistry system, using quality control material.

Measurand	Method	Unit	Intra‐assay CV (%)	Inter‐assay CV (%)	Low mean concentration	High mean concentration
β‐hydroxybutyrate	Enzymatic determination with β‐hydroxybutyrate dehydrogenase	μmol/L	0.36	1.39	262	3090
Non‐esterified fatty acids	Enzymatic endpoint	mmol/L	1.05	1.07	0.61	1.38
Triglycerides	Fossati enzymatic reaction with Trinder endpoint	mg/dL	0.20	0.40	88	177
Cholesterol	Enzymatic determination with Trinder endpoint	mg/dL	0.60	0.80	107	243
Total bilirubin	Vanadate oxidation	mg/dL	0.40	1.00	0.8	4.8
Aspartate aminotransferase	IFCC method	U/L	1.50	2.80	61	216
γ‐glutamyl transferase	Modified IFCC method	U/L	2.10	2.80	58	162
Total protein	Biuret method	g/dL	0.40	1.30	4.5	7.3
Albumin	Colorimetry—bromocresol green dye binding	g/dL	1.00	1.80	2.7	4.0
Urea nitrogen	Roch‐Ramel enzymatic reaction	mg/dL	1.00	2.20	16	50
Creatinine	Enzymatic determination with creatininase	mg/dL	0.50	0.90	0.46	5.19

For the simultaneous determination of 3‐MH and 1‐methylhistidine (1‐MH) concentrations, plasma samples were analyzed using hydrophilic interaction ultra‐high performance liquid chromatography coupled to tandem mass‐spectrometry (HPLC‐MS/MS). The development, validation, and quality control of the analytical method have been described in detail [12]. Briefly, recovery rates for 3‐MH were 78.5% (low spike), 84.0% (medium spike), and 91.8% (high spike), while for 1‐MH they were 84.9%, 79.3%, and 87.3% at the corresponding levels. Precision, expressed as relative standard deviation (RSD), remained < 10% for both isomers, with RSD values for 3‐MH ranging from 1.85% to 5.0% and for 1‐MH from 5.53% to 9.33% across the spiked concentration levels [12].

Available records of analytes for each study day were as follows: BHB, NEFA, TP, ALB, GLOB, BUN, CRE, 3‐MH, and 1‐MH on −21 days and on 21 days; AST, GGT, TRIG, CHOL, tBIL, BHB, NEFA, TP, ALB, GLOB, BUN, CRE, 3‐MH, and 1‐MH on −7 days and on 7 days; TP, ALB, GLOB, BUN, CRE, 3‐MH, and 1‐MH on 28 days.

Reference intervals for each study‐day were computed using the Reference Value Advisor (RefValAdv, v.2.1) freeware [5], following the guidelines set by the American Society for Veterinary Clinical Pathology [6]. Data distribution was visually examined with histograms and assessed statistically with Dixon‐Reed's and Tukey's tests. Outliers detected by either test were removed prior to RI computation only for the specific analyte at each time‐point, while “suspected” outliers were retained. The threshold for interpreting the *p*‐value of the Anderson‐Darling test for normal distribution was set at < 0.20 [17].

## 3 Results

From the original population of 238 multiparous Holstein cows, 113 cows met the inclusion criteria for the calculation of RIs. Although 113 cows were followed throughout the study, sample numbers varied per time point due to omissions, mislabeling, and practical constraints. Variations in analyte numbers per cow within the same time point were likely due to technical issues, insufficient sample volume, or automatic rejection of results failing quality control. Detailed distribution of cows per farm and per parity is provided in the Supporting Information S1. These cows were at a mean (±SD) parity of 2.7 (±0.8) and had a mean (±SD) dry period length of 60.5 days (±24.0). Mean BCS at −21 days was 3.28 (±0.46), 3.32 (±0.43) at −7 days, 3.07 (±0.40) at 7 days, 2.92 (±0.40) at 21 days, and 2.87 (±0.41) at 28 days. The mean standardized 305d milk yield for the subsequent lactation was 10,359 L (±2555). The excluded 125 cows were diagnosed with at least one case of abnormal rumen function (*n* = 25); fever (*n* = 75); lameness (*n* = 49); retained fetal membranes (*n* = 32); metritis (*n* = 67); mastitis (*n* = 12); left displaced abomasum (*n* = 8); ketosis (*n* = 7); milk fever (*n* = 2), and pneumonia (*n* = 2), while 12 cows were involuntarily culled before the end of the study period. No cows in our study required human assistance or intervention during calving.

**FIGURE 1:**
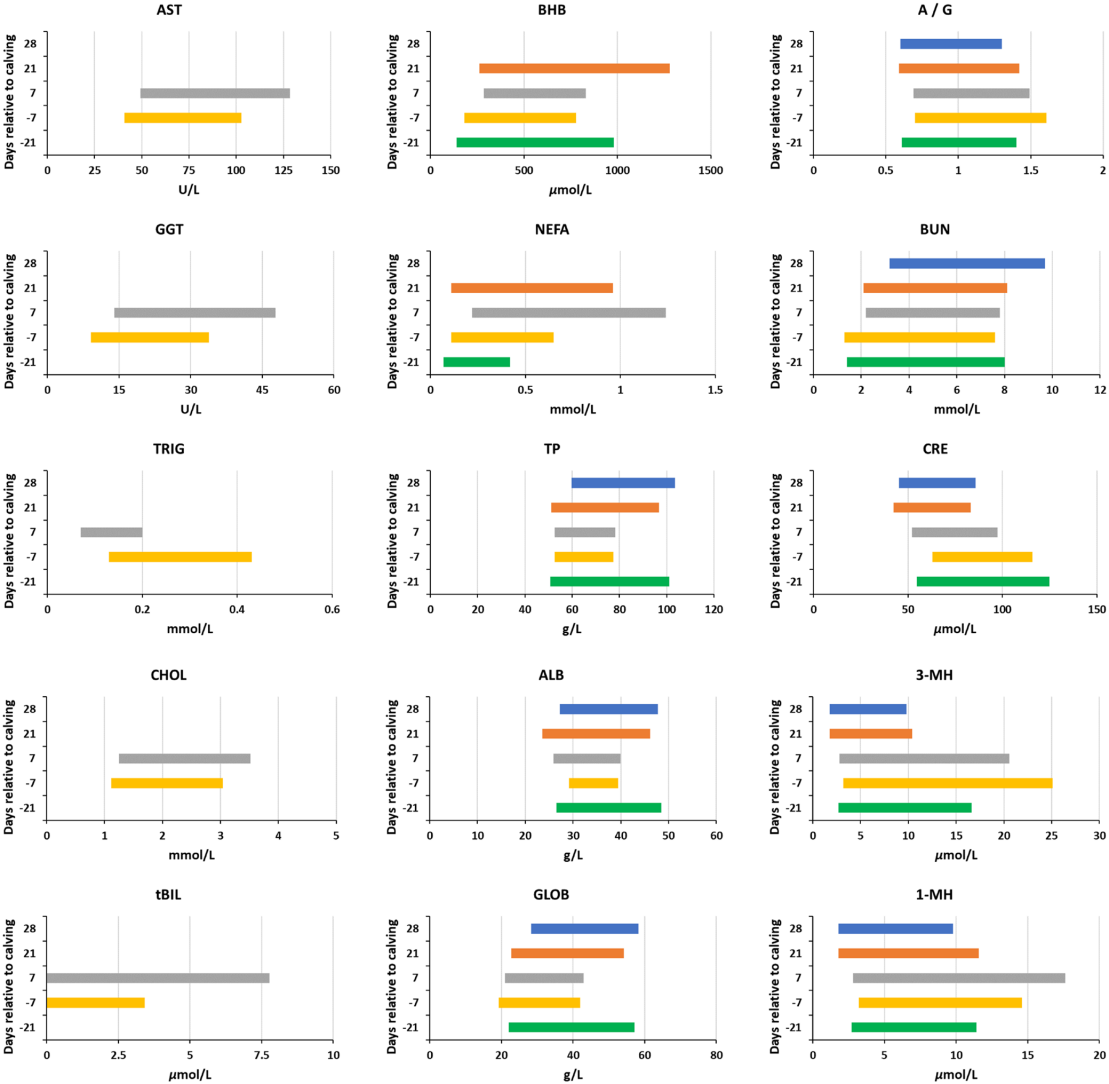
Reference intervals for serum aspartate aminotransferase (AST), γ‐glutamyl transferase (GGT), triglycerides (TRIG), cholesterol (CHOL), total bilirubin (tBIL), β‐hydroxybutyrate (BHB), non‐esterified fatty acids (NEFA), total protein (TP), albumin (ALB), globulin (GLOB), albumin/globulin ratio (A/G), urea nitrogen (BUN), creatinine (CRE), and for plasma 3‐methylhistidine (3‐MH) and 1‐methylhistidine (1‐MH), in 113 multiparous Holstein cows that remained clinically healthy throughout the periparturient period.

Computed RIs with 90% CIs for various serum and plasma biochemical parameters in clinically healthy cows at specific timepoints across the transition period are shown in detail in Tables 2, 3, 4, 5, 6 and summarized in Figure 1. Details about the removal of outliers, symmetry test, and characterization of CIs relative to the width of the RIs according to IFCC‐CLSI recommendations [6], are provided in the Supporting Information S1. To assess the potential effect of the farm on the variability of each analyte, we performed linear mixed‐effects models with repeated measurements, accounting for the random effect of the cow. A statistically significant (*p* < 0.05) farm effect was observed for all analytes except NEFA (*p* = 0.080) and tBIL (*p* = 0.203).

**TABLE 2:** Reference intervals (RIs) for serum and plasma biochemical analytes in clinically healthy multiparous Holstein cows at 21 days (±2 days) before expected calving day.

Measurand	Sample type	SI Units	Descriptives						RI computation						
			*N*	Mean	SD	Median	Min	Max	*p*	Distribution	Method	LRL of RI	URL of RI	CI 90% of LRL	CI 90% of URL
BHB	Serum	μmol/L	110	491.0	185.9	480.5	102.3	1013.2	0.117	NG	NP	141.0	981.9	102.3–221.3	828.1–1013.2
NEFA	Serum	mmol/L	107	0.18	0.09	0.16	0.02	0.46	< 0.001	NG	NP	0.07	0.42	0.02–0.09	0.37–0.46
TP	Serum	g/L	110	72.1	12.7	70.6	49.6	104.4	0.050	NG	BCTS	50.8	101.1	48.5–53.0	96.2–106.3
ALB	Serum	g/L	110	35.5	5.4	35.0	22.3	55.5	0.069	NG	NP	26.5	48.5	22.3–28.0	43.4–55.5
GLOB	Serum	g/L	110	36.6	9.0	36.1	21.2	62.3	< 0.001	NG	BCTS	22.1	57.2	20.7–23.5	53.5–60.9
A/G	Serum	—	110	1.01	0.2	1.00	0.56	1.51	0.940	G	US	0.61	1.40	0.56–0.66	1.35–1.45
BUN	Serum	mmol/L	110	4.6	1.6	4.6	1.1	8.7	0.840	G	US	1.4	8.0	1.0–1.8	7.5–8.2
CRE	Serum	μmol/L	110	82.2	16.9	81.3	49.5	131.8	0.004	NG	NP	54.8	124.7	49.5–60.1	115.0–131.7
3‐MH	Plasma	μmol/L	106	7.4	3.6	6.5	2.5	22.2	< 0.001	NG	NP	2.7	16.6	2.5–3.4	14.3–22.2
1‐MH	Plasma	μmol/L	107	5.8	2.3	5.7	2.1	13.6	< 0.001	NG	BCTS	2.5	11.23	2.2–2.8	10.3–12.3

Abbreviations: 1‐MH, 1‐methylhistidine; 3‐MH, 3‐methylhistidine; A/G, albumin/globulin ratio; ALB, albumin; BCTS, standard method on Box‐Cox transformed data; BHB, β‐hydroxybutyrate; BUN, urea nitrogen; CI, confidence interval; CRE, creatinine; G, Gaussian; GLOB, globulin; LRL, lower reference limit; NEFA, non‐esterified fatty acids; NG, non‐Gaussian; NP, non‐parametric; TP, total protein; URL, upper reference limit; US, standard method on untransformed data.

**TABLE 3:** Reference intervals (RIs) for serum and plasma biochemical analytes in clinically healthy multiparous Holstein cows at 7 days (±2 days) before expected calving day.

Measurand	Sample type	SI Units	Descriptives						RI computation						
			*N*	Mean	SD	Median	Min	Max	*p*	Distribution	Method	LRL of RI	URL of RI	CI 90% of LRL	CI 90% of URL
AST	Serum	U/L	93	63.3	14.3	61.1	36.0	114.0	< 0.001	NG	NP	40.9	102.8	36.0–43.7	90.1–114.0
GGT	Serum	U/L	93	21.4	6.2	21.4	6.3	40.0	0.844	G	US	9.1	33.8	7.4–10.8	32.0–35.6
TRIG	Serum	mmol/L	94	0.28	0.08	0.28	0.14	0.46	0.330	G	US	0.13	0.43	0.11–0.15	0.41–0.46
CHOL	Serum	mmol/L	94	2.07	0.48	2.11	0.80	3.13	0.741	G	US	1.11	3.04	0.98–1.24	2.90–3.17
tBIL	Serum	μmol/L	92	1.19	0.94	1.71	0.00	3.42	< 0.001	NG	NP	0.0	3.42	0.00–0.00	1.72–3.42
BHB	Serum	μmol/L	94	480.0	149.6	476.4	165.4	893.7	0.521	G	US	181.3	778.6	141.7–222.7	735.1–820.4
NEFA	Serum	mmol/L	89	0.25	0.13	0.21	0.06	0.69	< 0.001	NG	NP	0.11	0.65	0.06–0.12	0.52–0.69
TP	Serum	g/L	93	65.0	6.2	65.1	50.8	80.7	0.967	G	US	52.6	77.4	51.0–54.4	75.6–79.1
ALB	Serum	g/L	94	34.3	2.5	34.5	27.7	39.1	0.122	NG	NP	29.2	39.4	28.6–29.9	38.7–40.1
GLOB	Serum	g/L	94	30.5	5.3	30.3	18.5	41.4	0.448	G	US	19.4	42.0	18.5–21.4	39.5–42.5
A/G	Serum	—	93	1.16	0.23	1.14	0.59	1.81	0.378	G	US	0.70	1.61	0.64–0.77	1.54–1.67
BUN	Serum	mmol/L	94	4.4	1.6	4.6	1.3	7.7	0.173	NG	NP	1.3	7.6	0.9–1.7	7.1–8.0
CRE	Serum	μmol/L	94	89.3	13.3	88.4	63.7	128.2	0.319	G	US	62.8	115.8	59.2–66.3	111.4–119.4
3‐MH	Plasma	μmol/L	96	9.5	5.4	7.9	2.8	28.7	< 0.001	NG	BCTS	3.2	25.1	2.8–3.6	20.8–30.7
1‐MH	Plasma	μmol/L	96	6.6	2.8	6.1	2.3	15.0	< 0.001	NG	NP	2.7	14.1	2.3–3.1	12.8–15.0

Abbreviations: 1‐MH, 1‐methylhistidine; 3‐MH, 3‐methylhistidine; A/G, albumin/globulin ratio; ALB, albumin; AST, aspartate aminotransferase; BCTS, standard method on Box‐Cox transformed data; BHB, β‐hydroxybutyrate; BUN, urea nitrogen; CHOL, cholesterol; CI, confidence interval; CRE, creatinine; G, Gaussian; GGT, γ‐glutamyl transferase; GLOB, globulin; LRL, lower reference limit; NEFA, non‐esterified fatty acids; NG, non‐Gaussian; NP, non‐parametric; tBIL, total bilirubin; TP, total protein; TRIG, triglycerides; URL, upper reference limit; US, standard method on untransformed data.

**TABLE 4:** Reference intervals (RIs) for serum and plasma biochemical analytes in clinically healthy multiparous Holstein cows at 7 days (±2 days) after calving.

Measurand	Sample type	SI Units	Descriptives						RI computation						
			*N*	Mean	SD	Median	Min	Max	*p*	Distribution	Method	LRL of RI	URL of RI	CI 90% of LRL	CI 90% of URL
AST	Serum	U/L	98	79.9	19.4	76.8	46.4	138.2	< 0.001	NG	NP	49.3	128.5	46.4–50.6	120.7–138.2
GGT	Serum	U/L	99	25.4	7.7	24.6	10.6	52.1	0.001	NG	NP	14.0	47.8	10.6–15.8	38.9–52.1
TRIG	Serum	mmol/L	96	0.13	0.03	0.13	0.05	0.21	0.143	NG	NP	0.07	0.20	0.05–0.09	0.19–0.21
CHOL	Serum	mmol/L	99	2.10	0.51	2.04	0.98	3.75	0.035	NG	NP	1.25	3.52	0.98–1.41	3.01–3.75
tBIL	Serum	μmol/L	97	2.36	1.66	1.71	0.00	8.55	< 0.001	NG	NP	0.00	7.78	0.00–0.00	5.13–8.55
BHB	Serum	μmol/L	93	514.1	138.2	484.4	237.1	871.5	0.004	NG	NP	285.0	831.4	237.1–324.1	769.6–871.5
NEFA	Serum	mmol/L	99	0.62	0.26	0.57	0.21	1.36	0.005	NG	BCTS	0.22	1.24	0.19–1.24	1.12–1.36
TP	Serum	g/L	100	65.0	6.4	65.2	46.6	82.8	0.086	NG	NP	52.5	78.3	50.5–54.7	76.3–80.3
ALB	Serum	g/L	99	33.8	3.5	34.0	23.7	40.1	0.322	G	BCTS	25.9	39.9	24.3–27.4	39.2–40.6
GLOB	Serum	g/L	99	31.2	5.1	31.1	17.8	49.4	0.538	G	NP	20.80	43.20	17.8–23.7	3.89–49.4
A/G	Serum	—	99	1.11	0.21	1.08	0.68	1.95	0.207	NG	NP	0.69	1.49	0.68–0.81	1.43–1.95
BUN	Serum	mmol/L	99	4.7	1.4	4.6	1.1	8.9	0.787	G	NP	2.2	7.8	1.1–2.4	6.9–8.9
CRE	Serum	μmol/L	99	73.4	11.5	72.5	45.1	103.5	0.676	G	NP	52.2	97.3	45.1–56.6	92.8–103.5
3‐MH	Plasma	μmol/L	99	9.0	4.5	8.6	1.1	21.2	< 0.001	NG	NP	2.8	20.6	1.1–3.5	18.6–21.2
1‐MH	Plasma	μmol/L	100	6.4	3.0	6.2	0.9	15.3	< 0.001	NG	NP	2.3	14.8	0.9–2.8	13.1–15.3

Abbreviations: 1‐MH, 1‐methylhistidine; 3‐MH, 3‐methylhistidine; A/G, albumin/globulin ratio; ALB, albumin; AST, aspartate aminotransferase; BCTS, standard method on Box‐Cox transformed data; BHB, β‐hydroxybutyrate; BUN, urea nitrogen; CHOL, cholesterol; CI, confidence interval; CRE, creatinine; G, Gaussian; GGT, γ‐glutamyl transferase; GLOB, globulin; LRL, lower reference limit; NEFA, non‐esterified fatty acids; NG, non‐Gaussian; NP, non‐parametric; tBIL, total bilirubin; TP, total protein; TRIG, triglycerides; URL, upper reference limit.

**TABLE 5:** Reference intervals (RIs) for serum and plasma biochemical analytes in clinically healthy multiparous Holstein cows at 21 days (±2 days) after calving.

Measurand	Sample type	SI Units	Descriptives						RI computation						
			*N*	Mean	SD	Median	Min	Max	*p*	Distribution	Method	LRL of RI	URL of RI	CI 90% of LRL	CI 90% of URL
BHB	Serum	μmol/L	90	592.1	225.9	542.2	262.0	1335.8	< 0.001	NG	NP	262.0	1279.3	262.0–308.2	940.9–1335.8
NEFA	Serum	mmol/L	96	0.39	0.21	0.34	0.09	1.00	< 0.001	NG	BCTS	0.11	0.96	0.10–0.13	0.83–1.11
TP	Serum	g/L	98	70.7	11.5	70.3	51.0	101.9	0.072	NG	BCTS	51.2	96.8	49.0–53.4	92.2–101.8
ALB	Serum	g/L	98	34.8	5.6	34.8	22.8	49.1	0.579	G	US	23.6	46.1	22.1–25.1	44.5–47.6
GLOB	Serum	g/L	98	35.8	7.9	35.6	21.3	59.9	0.124	NG	BCTS	22.8	54.2	21.4–24.2	50.9–57.9
A/G	Serum	—	98	1.00	0.21	0.99	0.61	1.48	0.592	G	US	0.59	1.42	0.54–0.65	1.36–1.48
BUN	Serum	mmol/L	98	5.1	1.4	5.1	1.9	9.2	0.843	G	NP	2.1	8.1	1.9–2.8	7.7–9.2
CRE	Serum	μmol/L	98	62.8	10.6	61.9	41.6	89.3	0.360	G	US	42.4	83.1	38.0–43.1	76.8–81.9
3‐MH	Plasma	μmol/L	99	4.9	2.0	4.8	1.7	13.3	0.005	NG	NP	1.8	10.4	1.7–2.1	8.5–13.3
1‐MH	Plasma	μmol/L	101	5.4	2.4	5.0	1.4	13.4	0.004	NG	NP	1.9	10.5	1.4–2.2	9.9–13.4

Abbreviations: 1‐MH, 1‐methylhistidine; 3‐MH, 3‐methylhistidine; A/G, albumin/globulin ratio; ALB, albumin; BCTS, standard method on Box‐Cox transformed data; BHB, β‐hydroxybutyrate; BUN, urea nitrogen; CI, confidence interval; CRE, creatinine; G, Gaussian; GLOB, globulin; LRL, lower reference limit; NEFA, non‐esterified fatty acids; NG, non‐Gaussian; NP, non‐parametric; TP, total protein; URL, upper reference limit; US, standard method on untransformed data.

**TABLE 6:** Reference intervals (RIs) for serum and plasma biochemical analytes in clinically healthy multiparous Holstein cows at 28 days (±2 days) after calving.

Measurand	Sample type	SI Units	Descriptives						RI computation						
			*N*	Mean	SD	Median	Min	Max	*p*	Distribution	Method	LRL of RI	URL of RI	CI 90% of LRL	CI 90% of URL
TP	Serum	g/L	104	80.8	10.2	79.2	50.2	110.6	0.035	NG	NP	59.7	103.5	50.2–67.2	96.9–110.6
ALB	Serum	g/L	104	38.8	4.8	39.5	24.0	51.3	0.177	NG	NP	27.2	47.8	24.0–31.2	44.8–51.3
GLOB	Serum	g/L	104	42.0	7.8	40.5	26.0	67.9	0.050	NG	NP	28.3	58.3	26.0–30.9	55.3–67.9
A/G	Serum	—	104	0.95	0.18	0.95	0.57	1.36	0.851	G	US	0.60	1.30	0.55–0.64	1.25–1.35
BUN	Serum	mmol/L	104	5.9	1.6	5.8	2.5	11.4	0.141	NG	BCTS	3.2	9.7	2.9–3.5	9.0–9.6
CRE	Serum	μmol/L	104	65.4	9.7	64.5	43.3	91.1	0.131	NG	NP	45.1	85.8	40.5–45.6	78.5–84.4
3‐MH	Plasma	μmol/L	105	4.5	1.9	4.2	0.9	10.9	< 0.001	NG	NP	1.8	9.8	0.9–2.0	8.7–10.9
1‐MH	Plasma	μmol/L	105	5.0	2.4	4.7	1.4	14.3	0.001	NG	NP	1.9	11.7	1.4–2.0	9.2–14.3

Abbreviations: 1‐MH, 1‐methylhistidine; 3‐MH, 3‐methylhistidine; A/G, albumin/globulin ratio; ALB, albumin; BCTS, standard method on Box‐Cox transformed data; BHB, β‐hydroxybutyrate; BUN, urea nitrogen; CI, confidence interval; CRE, creatinine; G, Gaussian; GLOB, globulin; LRL, lower reference limit; NEFA, non‐esterified fatty acids; NG, non‐Gaussian; NP, non‐parametric; TP, total protein; URL, upper reference limit; US, standard method on untransformed data.

## 4 Discussion

Pre‐calving upper limits for NEFAs in our study were lower than those reported for cows at 8 to 1 day pre‐calving (1.00 mmol/L) [7] and at 20 to 10 days pre‐calving (0.76 mmol/L) [8]. Differences can be attributed to several reasons. Although a thorough clinical examination was performed in these studies to exclude cows with any clinical disease, these were followed until 7 days after calving [7] and 10 days before expected calving [8], while cows in our study were closely monitored up to 28 days after calving. Time of sampling relative to feeding is also a known factor that could alter NEFA concentrations [4]. The use of different assay methods across studies is another reasonable explanation for the observed differences. Interestingly, the widely used pre‐calving threshold of 0.4 mmol/L [18] matches with the upper limit at −21 days in our study, while the higher proposed thresholds of 0.5 [19] and 0.6 mmol/L [20] match better with the upper RI limit we found at −7 days. Post‐calving NEFA concentrations are also associated with reproduction and clinical diseases [21]; however, optimal values related to these outcomes remain a controversial topic. Upper RI limits at 7 and 21 days in our study were higher than the thresholds reported in the literature [21, 22]. Pre‐calving RIs for BHB in our study are similar to those reported for heifers and dry cows at 8 to 1 day pre‐calving [7]. The upper limit for BHB at 7 days in our study (831.4 μmol/L) was lower than that reported for cows at 3 days after calving (1710 μmol/L) [9] and for cows at 0–7 days after calving (1177 μmol/L) [7]. The latter referred studies reported an upper limit that either surpassed [9] or was close [7] to the commonly used threshold (1200 μmol/L) for subclinical ketosis [19], while a lower threshold (960.5 μmol/L) has been linked to increased risk of displaced abomasum, ketosis, metritis, and retained fetal membranes [21]. Depending on the timing of sampling, upper RIs may fall below the established thresholds, and clinicians should be cautious when interpreting BHB results. The possibility of week‐specific thresholds should not be dismissed and warrants investigation. Pre‐calving RIs for TRIG in our study are consistent with those previously reported in dry cows [8, 23]. Post‐calving RIs were comparable to those reported for early lactation cows (25–80 days‐in‐milk) [23] and notably lower and with a narrower range than the pre‐calving period.

Computed RIs for CHOL in our study are comparable to those previously reported in pre‐calving [7, 8] and post‐calving cows [7, 9]. Concentrations of CHOL decrease during late gestation as dry matter intake is low and low‐fat diets are provided, reaching a nadir of approximately 1.8 [4] or 2.2–2.5 mmol/L [24] at parturition, and increase thereafter as dry matter intake increases into lactation [10, 23].

The upper limit of tBIL at 7 days was more than 2‐fold greater than that at −7 days, but still below 8.55 μmol/L, concentrations above which are considered indicative of hepatic failure [4]. The spike in tBIL around parturition has been previously reported and has been attributed to the lower synthesis of the enzymes responsible for tBIL clearance [23].

Pre‐calving (−7 days) upper limit for AST in our study was approximately 16% higher (102.8 vs. 88.5 U/L) than that of dry cows and late‐pregnant heifers at 60 to 10 days before calving [8]. Its concentrations increased about 20% post‐calving, and the upper limit at 7 days was intermediate to those reported for cows at 3 ± 1 days and 30 ± 3 days post‐calving [9]. Since AST is also involved in amino acid metabolism, the post‐calving rise could also be due to the increased muscle protein and dipeptide catabolism occurring at this stage [25].

Our pre‐calving RIs for GGT are in accordance with those reported in dry cows [8, 23]. Similarly to AST, GGT activity increased post‐calving. Post‐calving (7 days), the upper limit in our study is similar to that reported in lactating cows at 30 days post‐calving [9] and at 25–80 days post‐calving [23], but much higher than that in cows at 3 days post‐calving.

The RIs for TP at −21 days in our study are wider than but comparable to those reported in dry cows and late‐pregnant heifers at 58–5 days [24], and at 60–10 days before calving [8]. Both those studies observed a parity effect with increased TP values in multiparous cows compared to primiparous ones and heifers [8, 24]. Computed lower limits in our study were similar to those reported in the literature and always above the cut‐point of 50 g/L, which would suggest protein deficiency or protein loss [4]. The lower TP values around calving reflect the state of negative protein balance, causing an uptake of labile amino acid reserves in blood, while higher values at −21 days and at 21 days reflect adequate protein nutrition at these stages.

Compared with the ALB RIs reported for dry cows and late‐pregnant heifers at 60–10 days pre‐calving [8] and at 3 ± 1 days and 30 ± 3 days post‐calving [9], lower limits computed in this study were 10%–20% lower. In our study, upper RI limits for GLOB fluctuated during the transition period, plummeted at −7 and 7 days, and stabilized again at 21 and 28 days at levels similar to those at −21 days. Our findings share similarities with the previously reported RIs in cows at 3 ± 1 days and 30 ± 3 days post‐calving [9]. Although the A/G ratio is generally considered stable in healthy cows, with a narrow RI below 1.0 [3], in our study we observed a wide range and the RIs were distributed almost symmetrically around 1.0, with the mean values ranging from 0.95 to 1.16 during the transition period, consistent with the previously reported RIs of 0.71–1.90 and 0.46–1.52 in cows at 3 ± 1 days and 30 ± 3 days post‐calving, respectively [9].

Previously reported BUN RIs for late‐pregnant heifers and dry cows at 60–10 days before calving were 1.1–5.8 mmol/L [8], and 2.1–8.0 mmol/L for dry cows at 8–1 days pre‐calving [7], with the latter being closer to the pre‐calving RIs computed in our population. Concentrations < 2.85 mmol/L or > 5.71 mmol/L indicate insufficient or excessive dietary protein intake [4]. RIs at 7 and 21 days in our study are consistent with those reported in cows at 0–7 days [7], and at 3 ± 1 days and at 30 ± 3 days post‐calving [9].

In our study, the upper CRE RI limits were decreased from −21 to 21 days, consistent with the mobilization of muscle protein and reduction in muscle thickness commonly observed during the transition period [25]. Prepartum RIs in our study are similar to those reported in pre‐calving heifers and dry cows 60–10 days before calving [8]. On average, post‐calving RIs in our study are lower than those reported for cows at 3 ± 1 days and 30 ± 3 days post‐calving [9] by 25%, but the declining rate through the first 4 weeks of the lactation is similar.

In the present study, the lower limit for 3‐MH remained unchanged during the entire transition period. The upper limit was increased from −21 to −7 days by approximately 50% and decreased gradually thereafter, reaching a low plateau at 21 and 28 days, at levels almost half of those at the beginning of the study period (−21 days). The increased upper limits at −7 and 7 days coincided with the decrease in ALB upper limits. From these results, we can reasonably infer that negative protein balance and myofibrillar protein degradation, particularly, peak 1 week before calving but are limited to the early post‐calving period only. The fact that the upper RI limit at −21 days was greater than those at 21 and 28 days does not necessarily imply that this muscle tissue catabolism was already initiated. We consider it is more likely indicative of higher muscle protein turnover in cows accreting muscle mass, as shown by the increase in the *longissimus dorsi* muscle thickness in most cows [25].

Aligned with 3‐MH, the lower limits for 1‐MH remained mostly unchanged, ranging from 1.9 to 2.7 μmol/L. The upper limits increased gradually from 21 days, peaked at 7 days, remaining always lower than the respective upper limits of 3‐MH, and decreased thereafter. This fluctuation was unexpected as the overall estimated marginal means for 1‐MH remained unaffected by time when accounting for various farm‐ and cow‐level factors [11]. From these results, we can infer that clinically healthy cows are expected to have an increase in 1‐MH concentrations after calving, contrary to the pre‐calving peak in 3‐MH concentrations.

## Conflicts of Interest

The authors declare no conflicts of interest.

## Supporting information


**Data S1:** vcp70044‐sup‐0001‐TableS1.docx.
