# Supplementary material for: Reference Intervals of Selected Serum and Plasma Biochemical Analytes in Clinically Healthy Multiparous Holstein Cows During the Periparturient Period
Source: Vet Clin Pathol. 2025 Sep 29;54(4):447–57. doi: 10.1111/vcp.70044 (PMC12885868; doi:10.1111/vcp.70044)
Supplement: Supplementary file 1 — Data S1: vcp70044‐sup‐0001‐TableS1.docx. [file VCP-54-447-s001.docx]

**Reference intervals for selected serum and plasma biochemical analytes in clinically healthy multiparous Holstein cows during the periparturient period**

**Running title: BIOCHEMICAL REFERENCE INTERVALS IN TRANSITION COWS**

**Supplemental Material.**

**Supplemental Table 1**. Distribution of cows meeting the inclusion and exclusion criteria for the calculation of reference intervals per farm and per parity.

| Parity | Farm A | Farm B | Farm C | Farm D | Farm E | Farm F | Total n |
| --- | --- | --- | --- | --- | --- | --- | --- |
| 2^nd^ | 13 | 7 | 4 | 5 | 22 | 7 | 56 |
| 3^rd^ | 3 | 8 | 7 | 7 | 7 | 5 | 37 |
| 4^th^ | 2 |  | 1 | 1 | 4 |  | 8 |
| 5^th^ | 3 |  |  | 1 | 1 | 1 | 6 |
| 6^th^ | 3 |  | 1 | 1 |  |  | 5 |
| 7^th^ |  |  |  |  | 1 |  | 1 |
| Total n | 24 | 15 | 13 | 15 | 35 | 13 | 113 |

**Supplemental Table 2**. Reference intervals (RIs) for serum and plasma biochemical analytes in clinically healthy multiparous Holstein cows at 21 days (± 2 days) before expected calving day.

| **Measurands** | | | **Descriptive Statistics** | | | | | | | | **RI Computation** | | | | | | **RI Characterization** | | |
| --- | --- | --- | --- | --- | --- | --- | --- | --- | --- | --- | --- | --- | --- | --- | --- | --- | --- | --- | --- |
| **Measurand** | **Conventional Units** | **SI Units** | **Initial n** | **Removed outliers** | **Final n** | **Mean** | **SD** | **Median** | **Min** | **Max** | **Normality test p-value** | **Symmetry test p-value** | **Distribution** | **Method** | **LRL of RI^g^** | **URL of RI** | **0.2 width of RI** | **CI 90% of LRL** | **CI 90% of URL** |
| BHB | mmol/L | mmol/L | 110 | 0 | 110 | 491.0 | 185.9 | 480.5 | 102.3 | 1013.2 | 0.117 | 0.386 | NG/S | NP | 141.0 | 981.9 | 168.2 | 102.3-221.3 | 828.1-1013.2 |
| NEFA | mmol/L | mmol/L | 110 | 3 | 107 | 0.18 | 0.09 | 0.16 | 0.02 | 0.46 | <0.001 | <0.001 | NG/NS | NP | 0.07 | 0.42 | 0.07 | 0.02-0.09 | 0.37-0.46 |
| TP | g/dL | g/L | 110 | 0 | 110 | 72.1 | 12.7 | 70.6 | 49.6 | 104.4 | 0.050 | 0.011 | NG/NS | BCTP | 50.8 | 101.1 | 10.1 | 48.5-53.0 | 96.2-106.3 |
| ALB | g/dL | g/L | 110 | 0 | 110 | 35.5 | 5.4 | 35.0 | 22.3 | 55.5 | 0.069 | 0.222 | NG/S | NP | 26.5 | 48.5 | 4.4 | 22.3-28.0 | 43.4-55.5 |
| GLOB | g/dL | g/L | 110 | 0 | 110 | 36.6 | 9.0 | 36.1 | 21.2 | 62.3 | <0.001 | 0.576 | NG/S | BCTP | 22.1 | 57.2 | 7.0 | 20.7-23.5 | 53.5-60.9 |
| A/G | NA | NA | 110 | 0 | 110 | 1.01 | 0.2 | 1.00 | 0.56 | 1.51 | 0.940 | 0.576 | G/S | UP | 0.61 | 1.40 | 0.16 | 0.56-0.66 | 1.35-1.45 |
| BUN | mg/dL | mmol/L | 110 | 0 | 110 | 4.6 | 1.6 | 4.6 | 1.1 | 8.7 | 0.840 | 0.910 | G/S | UP | 1.4 | 8.0 | 1.3 | 1.0-1.8 | 7.5-8.2 |
| CRE | mg/dL | µmol/L | 110 | 0 | 110 | 82.2 | 16.9 | 81.3 | 49.5 | 131.8 | 0.004 | 0.006 | NG/NS | NP | 54.8 | 124.7 | 14.1 | 49.5-60.1 | 115.0-131.7 |
| 3-MH | μmol/L | μmol/L | 109 | 3 | 106 | 7.4 | 3.6 | 6.5 | 2.5 | 22.2 | <0.001 | <0.001 | NG/NS | NP | 2.7 | 16.6 | 2.8 | 2.5-3.4 | 14.3-22.2 |
| 1-MH | μmol/L | μmol/L | 109 | 2 | 107 | 5.8 | 2.3 | 5.7 | 2.1 | 13.6 | <0.001 | 0.007 | NG/NS | BCTP | 2.5 | 11.23 | 1.8 | 2.2-2.8 | 10.3-12.3 |

BHB, β-hydroxybutyrate, NEFA, non-esterified fatty acids, TP, total protein, ALB, albumin, GLOB, globulin, A / G, albumin / globulin ratio, BUN, urea nitrogen, CRE, creatinine, 3-MH, 3-methylhistidine, 1-MH, 1-methylhistidine.

NG, non-Gaussian, G, Gaussian, NP, non-parametric, BCTP, parametric method on Box-Cox transformed data, UP, parametric method on untransformed data, LRL, lower reference limit, URL, upper reference limit, CI, confidence interval.

**Supplemental Table 3.** Reference intervals (RIs) for serum and plasma biochemical analytes in clinically healthy multiparous Holstein cows at 7 days (± 2 days) before expected calving day.

| **Measurands** | | | **Descriptive Statistics** | | | | | | | | **RI Computation** | | | | | | **RI Characterization** | | |
| --- | --- | --- | --- | --- | --- | --- | --- | --- | --- | --- | --- | --- | --- | --- | --- | --- | --- | --- | --- |
| **Measurand** | **Conventional Units** | **SI Units** | **Initial n** | **Removed outliers** | **Final n** | **Mean** | **SD** | **Median** | **Min** | **Max** | **Normality test p-value** | **Symmetry test p-value** | **Distribution** | **Method** | **LRL of RI^g^** | **URL of RI^g^** | **0.2 width of RI** | **CI 90% of LRL** | **CI 90% of URL** |
| AST | U/L | U/L | 94 | 1 | 93 | 63.3 | 14.3 | 61.1 | 36.0 | 114.0 | <0.001 | 0.541 | NG/S | NP | 40.9 | 102.8 | 12.4 | 36.0-43.7 | 90.1-114.0 |
| GGT | U/L | U/L | 94 | 1 | 93 | 21.4 | 6.2 | 21.4 | 6.3 | 40.0 | 0.844 | 0.826 | G/NS | UP | 9.1 | 33.8 | 5.0 | 7.4-10.8 | 32.0-35.6 |
| TRIG | mg/dL | mmol/L | 94 | 0 | 94 | 0.28 | 0.08 | 0.28 | 0.14 | 0.46 | 0.330 | 0.733 | G/S | UP | 0.13 | 0.43 | 0.06 | 0.11-0.15 | 0.41-0.46 |
| CHOL | mg/dL | mmol/L | 94 | 0 | 94 | 2.07 | 0.48 | 2.11 | 0.80 | 3.13 | 0.741 | 0.150 | G/S | UP | 1.11 | 3.04 | 0.39 | 0.98-1.24 | 2.90-3.17 |
| tBIL | mg/dL | µmol/L | 94 | 2 | 92 | 1.19 | 0.94 | 1.71 | 0.00 | 3.42 | <0.001 | <0.001 | NG/NS | NP | 0.00 | 3.42 | 0.68 | 0.00-0.00 | 1.71-3.42 |
| BHB | mmol/L | mmol/L | 95 | 1 | 94 | 480.0 | 149.6 | 476.4 | 165.4 | 893.7 | 0.521 | 0.150 | G/S | UP | 181.3 | 778.6 | 119.5 | 141.7-222.7 | 735.1-820.4 |
| NEFA | mmol/L | mmol/L | 94 | 5 | 89 | 0.25 | 0.13 | 0.21 | 0.06 | 0.69 | <0.001 | <0.001 | NG/NS | NP | 0.11 | 0.65 | 0.11 | 0.06-0.12 | 0.52-0.69 |
| TP | g/dL | g/L | 94 | 0 | 94 | 65.0 | 6.2 | 65.1 | 50.8 | 80.7 | 0.967 | 0.107 | G/S | UP | 52.6 | 77.4 | 5.0 | 51.0-54.4 | 75.6-79.1 |
| ALB | g/dL | g/L | 94 | 0 | 94 | 34.3 | 2.5 | 34.5 | 27.7 | 39.1 | 0.122 | <0.001 | NG/NS | UP | 29.2 | 39.4 | 2.0 | 28.6-29.9 | 38.7-40.1 |
| GLOB | g/dL | g/L | 94 | 1 | 93 | 30.5 | 5.3 | 30.3 | 18.5 | 41.4 | 0.448 | 0.874 | G/S | UP | 19.4 | 42.0 | 4.5 | 18.5-21.4 | 39.5-42.5 |
| A/G | NA | NA | 94 | 0 | 94 | 1.16 | 0.23 | 1.14 | 0.59 | 1.81 | 0.378 | 0.733 | G/S | UP | 0.70 | 1.61 | 0.18 | 0.64-0.77 | 1.54-1.67 |
| BUN | mg/dL | mmol/L | 94 | 0 | 94 | 4.4 | 1.6 | 4.6 | 1.3 | 7.7 | 0.173 | 0.994 | NG/S | UP | 1.3 | 7.6 | 1.2 | 0.9-1.7 | 7.1-8.0 |
| CRE | mg/dL | µmol/L | 93 | 0 | 93 | 89.3 | 13.3 | 88.4 | 63.7 | 128.2 | 0.319 | 0.008 | G/NS | UP | 62.8 | 115.8 | 10.6 | 59.2-66.3 | 111.4-119.4 |
| 3-MH | μmol/L | μmol/L | 97 | 1 | 96 | 9.5 | 5.4 | 7.9 | 2.8 | 28.7 | <0.001 | 0.050 | NG/S | BCTP | 3.2 | 25.1 | 4.4 | 2.8-3.6 | 20.8-30.7 |
| 1-MH | μmol/L | μmol/L | 97 | 1 | 96 | 6.6 | 2.8 | 6.1 | 2.3 | 15.0 | <0.001 | 0.004 | NG/NS | NP | 2.7 | 14.1 | 2.3 | 2.3-3.1 | 12.8-15.0 |

AST, aspartate aminotransferase, GGT, γ-glutamyl transferase, TRIG, triglycerides, CHOL, cholesterol, tBIL, total bilirubin, BHB, β-hydroxybutyrate, NEFA, non-esterified fatty acids, TP, total protein, ALB, albumin, GLOB, globulin, A / G, albumin / globulin ratio, BUN, urea nitrogen, CRE, creatinine, 3-MH, 3-methylhistidine, 1-MH, 1-methylhistidine.

NG, non-Gaussian, G, Gaussian, NP, non-parametric, UP, parametric method on untransformed data, BCTP, parametric method on Box-Cox transformed data, LRL, lower reference limit, URL, upper reference limit, CI, confidence interval.

**Supplemental Table 4.** Reference intervals (RIs) for serum and plasma biochemical analytes in clinically healthy multiparous Holstein cows at 7 days (± 2 days) after calving.

| **Measurands** | | | **Descriptive Statistics** | | | | | | | | **RI Computation** | | | | | | **RI Characterization** | | |
| --- | --- | --- | --- | --- | --- | --- | --- | --- | --- | --- | --- | --- | --- | --- | --- | --- | --- | --- | --- |
| **Measurand** | **Conventional Units** | **SI Units** | **Initial n** | **Removed outliers** | **Final n** | **Mean** | **SD** | **Median** | **Min** | **Max** | **Normality test p-value** | **Symmetry test p-value** | **Distribution** | **Method** | **LRL of RI** | **URL of RI** | **0.2 width of RI** | **CI 90% of LRL** | **CI 90% of URL** |
| AST | U/L | U/L | 99 | 1 | 98 | 79.9 | 19.4 | 76.8 | 46.4 | 138.2 | <0.001 | 0.729 | NG/S | NP | 49.3 | 128.5 | 15.8 | 46.4-50.6 | 120.7-138.2 |
| GGT | U/L | U/L | 99 | 0 | 99 | 25.4 | 7.7 | 24.6 | 10.6 | 52.1 | 0.001 | 0.760 | NG/S | NP | 14.0 | 47.8 | 6.8 | 10.6-15.8 | 38.9-52.1 |
| TRIG | mg/dL | mmol/L | 98 | 2 | 96 | 0.28 | 0.08 | 0.28 | 0.14 | 0.46 | 0.143 | 0.419 | NG/S | NP | 0.13 | 0.43 | 0.06 | 0.11-0.15 | 0.41-0.46 |
| CHOL | mg/dL | mmol/L | 99 | 0 | 99 | 2.10 | 0.51 | 2.04 | 0.98 | 3.75 | 0.035 | 0.017 | NG/NS | NP | 1.25 | 3.52 | 0.45 | 0.98-1.41 | 3.01-3.75 |
| tBIL | mg/dL | µmol/L | 98 | 1 | 97 | 2.36 | 1.66 | 1.71 | 0.00 | 8.55 | <0.001 | <0.001 | NG/NS | NP | 0.00 | 7.78 | 1.56 | 0.00-0.00 | 5.13-8.55 |
| BHB | mmol/L | mmol/L | 101 | 8 | 93 | 514.1 | 138.2 | 484.4 | 237.1 | 871.5 | 0.004 | 0.541 | NG/S | NP | 285.0 | 831.4 | 109.3 | 237.1-324.1 | 769.6-871.5 |
| NEFA | mmol/L | mmol/L | 99 | 0 | 99 | 0.62 | 0.26 | 0.57 | 0.21 | 1.36 | 0.005 | 0.017 | NG/NS | BCTP | 0.22 | 1.24 | 0.20 | 0.19-1.24 | 1.12-1.36 |
| TP | g/dL | g/L | 100 | 0 | 100 | 65.0 | 6.4 | 65.2 | 46.6 | 82.8 | 0.086 | 0.693 | NG/S | NP | 52.5 | 78.3 | 5.2 | 50.5-54.7 | 76.3-80.3 |
| ALB | g/dL | g/L | 99 | 0 | 99 | 33.8 | 3.5 | 34.0 | 23.7 | 40.1 | 0.322 | 0.381 | G/S | BCTP | 25.9 | 39.9 | 2.8 | 24.3-27.4 | 39.2-40.6 |
| GLOB | g/dL | g/L | 99 | 0 | 99 | 31.2 | 5.1 | 31.1 | 17.8 | 49.4 | 0.538 | 0.760 | G/S | NP | 20.80 | 43.20 | 4.5 | 17.8-23.7 | 3.89-49.4 |
| A/G | NA | NA | 99 | 0 | 99 | 1.11 | 0.21 | 1.08 | 0.68 | 1.95 | 0.207 | 0.094 | NG/S | NP | 0.69 | 1.49 | 0.16 | 0.68-0.81 | 1.43-1.95 |
| BUN | mg/dL | mmol/L | 99 | 0 | 99 | 4.7 | 1.4 | 4.6 | 1.1 | 8.9 | 0.787 | 0.619 | G/S | NP | 2.2 | 7.8 | 1.1 | 1.1-2.4 | 6.9-8.9 |
| CRE | mg/dL | µmol/L | 99 | 0 | 99 | 73.4 | 11.5 | 72.5 | 45.1 | 103.5 | 0.676 | 0.043 | G/NS | NP | 52.2 | 97.3 | 8.8 | 45.1-56.6 | 92.8-103.5 |
| 3-MH | μmol/L | μmol/L | 100 | 1 | 99 | 9.0 | 4.5 | 8.6 | 1.1 | 21.2 | <0.001 | 0.182 | NG/S | NP | 2.8 | 20.6 | 3.6 | 1.1-3.5 | 18.6-21.2 |
| 1-MH | μmol/L | μmol/L | 100 | 0 | 100 | 6.4 | 3.0 | 6.2 | 0.9 | 15.3 | <0.001 | <0.001 | NG/NS | NP | 2.3 | 14.8 | 2.5 | 0.9-2.8 | 13.1-15.3 |

AST, aspartate aminotransferase, GGT, γ-glutamyl transferase, TRIG, triglycerides, CHOL, cholesterol, tBIL, total bilirubin, BHB, β-hydroxybutyrate, NEFA, non-esterified fatty acids, TP, total protein, ALB, albumin, GLOB, globulin, A / G, albumin / globulin ratio, BUN, urea nitrogen, CRE, creatinine, 3-MH, 3-methylhistidine, 1-MH, 1-methylhistidine.

NG, non-Gaussian, G, Gaussian, NP, non-parametric, BCTP, parametric method on Box-Cox transformed data, LRL, lower reference limit, URL, upper reference limit, CI, confidence interval.

**Supplemental Table 5.** Reference intervals (RIs) for serum and plasma biochemical analytes in clinically healthy multiparous Holstein cows at 21 days (± 2 days) after calving.

| **Measurands** | | | **Descriptive Statistics** | | | | | | | | **RI Computation** | | | | | | **RI Characterization** | | |
| --- | --- | --- | --- | --- | --- | --- | --- | --- | --- | --- | --- | --- | --- | --- | --- | --- | --- | --- | --- |
| **Measurand** | **Conventional Units** | **SI Units** | **Initial n** | **Removed outliers** | **Final n** | **Mean** | **SD** | **Median** | **Min** | **Max** | **Normality test p-value** | **Symmetry test p-value** | **Distribution** | **Method** | **LRL of RI** | **URL of RI** | **0.2 width of RI** | **CI 90% of LRL** | **CI 90% of URL** |
| BHB | mmol/L | mmol/L | 100 | 10 | 90 | 592.1 | 225.9 | 542.2 | 262.0 | 1335.8 | <0.001 | 0.069 | NG/S | NP | 262.0 | 1279.3 | 203.5 | 262.0-308.2 | 940.9-1335.8 |
| NEFA | mmol/L | mmol/L | 98 | 2 | 96 | 0.39 | 0.21 | 0.34 | 0.09 | 1.00 | <0.001 | <0.001 | NG/NS | BCTP | 0.11 | 0.96 | 0.17 | 0.10-0.13 | 0.83-1.11 |
| TP | g/dL | g/L | 98 | 0 | 98 | 70.7 | 11.5 | 70.3 | 51.0 | 101.9 | 0.072 | 0.155 | NG/S | BCTP | 51.2 | 96.8 | 9.1 | 49.0-53.4 | 92.2-101.8 |
| ALB | g/dL | g/L | 98 | 0 | 98 | 34.8 | 5.6 | 34.8 | 22.8 | 49.1 | 0.579 | 0.580 | G/S | UP | 23.6 | 46.1 | 4.5 | 22.1-25.1 | 44.5-47.6 |
| GLOB | g/dL | g/L | 98 | 0 | 98 | 35.8 | 7.9 | 35.6 | 21.3 | 59.9 | 0.124 | 0.845 | NG/S | BCTP | 22.8 | 54.2 | 6.3 | 21.4-24.2 | 50.9-57.9 |
| A/G | NA | NA | 98 | 0 | 98 | 1.00 | 0.21 | 0.99 | 0.61 | 1.48 | 0.592 | 0.729 | G/S | UP | 0.59 | 1.42 | 0.17 | 0.54-0.65 | 1.36-1.48 |
| BUN | mg/dL | mmol/L | 98 | 0 | 98 | 5.1 | 1.4 | 5.1 | 1.9 | 9.2 | 0.843 | 0.988 | G/S | NP | 2.1 | 8.1 | 1.2 | 1.9-2.8 | 7.7-9.2 |
| CRE | mg/dL | µmol/L | 98 | 0 | 98 | 62.8 | 10.6 | 61.9 | 41.6 | 89.3 | 0.360 | 0.002 | G/NS | UP | 42.4 | 83.1 | 8.0 | 38.0-43.1 | 76.8-81.9 |
| 3-MH | μmol/L | μmol/L | 101 | 2 | 99 | 4.9 | 2.0 | 4.8 | 1.7 | 13.3 | 0.005 | 0.043 | NG/NS | NP | 1.8 | 10.4 | 1.7 | 1.7-2.1 | 8.5-13.3 |
| 1-MH | μmol/L | μmol/L | 102 | 1 | 101 | 5.4 | 2.4 | 5.0 | 1.4 | 13.4 | 0.004 | <0.001 | NG/NS | NP | 1.9 | 10.5 | 1.7 | 1.4-2.2 | 9.9-13.4 |

BHB, β-hydroxybutyrate, NEFA, non-esterified fatty acids, TP, total protein, ALB, albumin, GLOB, globulin, A / G, albumin / globulin ratio, BUN, urea nitrogen, CRE, creatinine, 3-MH, 3-methylhistidine, 1-MH, 1-methylhistidine.

NG, non-Gaussian, G, Gaussian, NP, non-parametric, BCTP, parametric method on Box-Cox transformed data, UP, parametric method on untransformed data, LRL, lower reference limit, URL, upper reference limit, CI, confidence interval.

**Supplemental Table 6.** Reference intervals (RIs) for serum and plasma biochemical analytes in clinically healthy multiparous Holstein cows at 28 days (± 2 days) after calving.

| **Measurands** | | | **Descriptive Statistics** | | | | | | | | **RI Computation** | | | | | | **RI Characterization** | | |
| --- | --- | --- | --- | --- | --- | --- | --- | --- | --- | --- | --- | --- | --- | --- | --- | --- | --- | --- | --- |
| **Measurand** | **Conventional Units** | **SI Units** | **Initial n** | **Removed outliers** | **Final n** | **Mean** | **SD** | **Median** | **Min** | **Max** | **Normality test p-value** | **Symmetry test p-value** | **Distribution** | **Method** | **LRL of RI** | **URL of RI** | **0.2 width of RI** | **CI 90% of LRL** | **CI 90% of URL** |
| TP | g/dL | g/L | 104 | 0 | 104 | 80.8 | 10.2 | 79.2 | 50.2 | 110.6 | 0.035 | 0.015 | NG/NS | NP | 59.7 | 103.5 | 8.8 | 50.2-67.2 | 96.9-110.6 |
| ALB | g/dL | g/L | 104 | 0 | 104 | 38.8 | 4.8 | 39.5 | 24.0 | 51.3 | 0.177 | 0.024 | NG/NS | NP | 27.2 | 47.8 | 4.1 | 24.0-31.2 | 44.8-51.3 |
| GLOB | g/dL | g/L | 104 | 0 | 104 | 42.0 | 7.8 | 40.5 | 26.0 | 67.9 | 0.050 | 0.578 | NG/NS | NP | 28.3 | 58.3 | 6.0 | 26.0-30.9 | 55.3-67.9 |
| A/G | NA | NA | 104 | 0 | 104 | 0.95 | 0.18 | 0.95 | 0.57 | 1.36 | 0.851 | 0.500 | G/S | UP | 0.60 | 1.30 | 0.14 | 0.55-0.64 | 1.25-1.35 |
| BUN | mg/dL | mmol/L | 104 | 0 | 104 | 5.9 | 1.6 | 5.8 | 2.5 | 11.4 | 0.141 | 0.723 | NG/S | BCTP | 3.2 | 9.7 | 1.3 | 2.9-3.5 | 9.0-9.6 |
| CRE | mg/dL | µmol/L | 104 | 0 | 104 | 65.4 | 9.7 | 64.5 | 43.3 | 91.1 | 0.131 | 0.015 | NG/NS | NP | 45.1 | 85.8 | 8.0 | 40.5-45.6 | 78.5-84.4 |
| 3-MH | μmol/L | μmol/L | 105 | 0 | 105 | 4.5 | 1.9 | 4.2 | 0.9 | 10.9 | <0.001 | 0.012 | NG/NS | NP | 1.8 | 9.8 | 1.6 | 0.9-2.0 | 8.7-10.9 |
| 1-MH | μmol/L | μmol/L | 105 | 0 | 105 | 5.0 | 2.4 | 4.7 | 1.4 | 14.3 | 0.001 | 0.004 | NG/NS | NP | 1.9 | 11.7 | 2.0 | 1.4-2.0 | 9.2-14.3 |

BHB, β-hydroxybutyrate, NEFA, non-esterified fatty acids, TP, total protein, ALB, albumin, GLOB, globulin, A / G, albumin / globulin ratio, BUN, urea nitrogen, CRE, creatinine, 3-MH, 3-methylhistidine, 1-MH, 1-methylhistidine.

NG, non-Gaussian, G, Gaussian, NP, non-parametric, UP, parametric method on untransformed data, BCTP, parametric method on Box-Cox transformed data, LRL, lower reference limit, URL, upper reference limit, CI, confidence interval.
